# Six years’ experience and trends of serum 25-hydroxy vitamin D concentration and the effect of vitamin D_3_ consumption on these trends

**DOI:** 10.3389/fphar.2023.1232285

**Published:** 2023-07-14

**Authors:** László Horváth, Sara Mirani, Michael Magdy Fahmy Girgis, Szilvia Rácz, Ildikó Bácskay, Harjit Pal Bhattoa, Béla E. Tóth

**Affiliations:** ^1^ Department of Pharmaceutical Surveillance and Economics, Faculty of Pharmacy, University of Debrecen, Debrecen, Hungary; ^2^ Department of Medical Imaging, Faculty of Medicine, University of Debrecen, Debrecen, Hungary; ^3^ Healthcare Industry Institute, University of Debrecen, Debrecen, Hungary; ^4^ Department of Pharmaceutical Technology, Faculty of Pharmacy, University of Debrecen, Debrecen, Hungary; ^5^ Department of Laboratory Medicine, Faculty of Medicine, University of Debrecen, Debrecen, Hungary

**Keywords:** vitamin D, drug utilization research, COVID-19, vitamin D supplementation, prevention

## Abstract

**Introduction:** Vitamin D (vitD) deficiency may have importance in some diseases, but there is a lack of data in our country to clarify the current situation. Our aim was to examine the basic characteristics of patients’ vitD status, and the ratio of vitD deficiency and its relation to certain diseases, assess seasonality and trends, and reveal the indirect impact of the COVID-19 pandemic on vitD_3_ supplementation at the patient population level.

**Methods:** Anonymized data on 25(OH)D test results were obtained from the clinical data registry of a tertiary teaching hospital covering the period between 1 January 2015 and 30 June 2021. VitD consumption (pharmacy sale) data were retrieved from the database of the National Health Insurance Fund of Hungary in order to calculate the defined daily dose (DDD)/1,000 inhabitants/day. Descriptive statistics and odds ratios with their 95% confidence intervals were calculated. The two-sample *t*-test and F-test were used to analyze our patients’ data. Significant differences were considered if *p* <0.05.

**Results:** Altogether, 45,567 samples were investigated; the mean age was 49 ± 19.1 years and 68.4% of them were female subjects. Overall, 20% of all patients had hypovitaminosis D, and just over 7% of patients had vitD deficiency. Male subjects had higher odds for hypovitaminosis or vitD deficiency (65.4 ± 28.2 nmol/L *vs.* 68.4 ± 28.4 nmol/L; *p* <0.0001). The mean 25(OH)D concentration has changed during the year, reaching a peak in September and a minimum in February. Patients with diseases of the circulatory system, genitourinary system, certain conditions originating in the perinatal period, and “sine morbo” (i.e., without a disease; such as those aged over 45 years and female teenagers) had statistically higher odds for lower 25(OH)D concentrations (*p* <0.00001). VitD consumption showed seasonality, being higher in autumn and winter. A slight increase started in the season of 2017/18, and two huge peaks were detected at the beginning of 2020 and 2021 in association with the COVID-19 waves.

**Conclusion:** Our data are the first to describe data concerning vitD in our region. It reinforces the notion of vitD_3_ supplementation for some risk groups and also in healthy individuals. To prevent the winter decline, vitD_3_ supplementation should be started in September. This and the results during the COVID-19 pandemic highlight the importance of health education encouraging vitamin D_3_ supplementation.

## Introduction

Vitamin D is an essential fat-soluble vitamin, which is synthesized in the skin, the process requiring sunshine and provitamin D_3_ (Norman, 2008). Its metabolite is measured in the blood serum known as 25-hydroxy vitamin D (25(OH)D), showing the vitamin D supply of the body. Despite the fact that exact biochemical pathways are unknown in many cases, the receptors of vitamin D on organs presume the role of 25(OH)D in the function of those organs. For example, it plays a role in the immune system and it is important in daily life as one of the primary biological regulators of calcium homeostasis. There is an individual’s responsiveness to various vitamin D_3_ doses based on their ability to convert it to its active metabolite and its interaction with receptor and response elements, which can be explained by genetic and epigenetic individual differences (Shirvani et al., 2019). It modulates multiple components of the innate and adaptive immune system and endothelial membrane stability, which is why its deficiency can lead to an increased risk of many immune-related diseases like psoriasis, multiple sclerosis, rheumatoid arthritis, and type 1 diabetes (Charoenngam and Holick, 2020). Activated 25(OH)D is also a steroid hormone (Norman, 2008). Despite the extensive differences between drugs and nutrients, the methods of evidence-based medicine (EBM) have recently been applied to studies concerning nutrients. Heaney developed a system for the standardization of clinical studies on nutrient effects that was based on the analysis of the typical sigmoid curve of biological response to nutrients (Heaney, 2014). One randomized field trial study on the screening and treatment program of prenatal vitamin D deficiency can be considered an example of few supplementation studies that are mostly based on Heaney’s criteria (Rostami et al., 2018).

Vitamin D is an essential dietary component which contributes to good health (traditionally bone health), with a major function in calcium and phosphate metabolism regulation, which maintains a healthy, mineralized skeleton, and its deficiency is linked to rickets in childhood; osteomalacia in adults; and osteoporosis aggravation, chronic musculoskeletal pain, muscle weakness, and predisposition to falls in the elderly (Charoenngam et al., 2019). Serum 25(OH)D concentrations show a seasonal change even in cases of hypovitaminosis D or vitamin D deficiency (Schöttker et al., 2014). The prevalence of vitamin D deficiency is moderate in Europe, as seen in the study by Cashman (2022). In that study, a pooled estimate was observed, using <30 and <50 nmol/L of 25(OH)D serum concentration thresholds; the prevalence of deficiency and insufficiency at the population level was 13% and 40.4%, respectively. In the last few decades, vitamin D deficiency has been associated with many diseases and mortalities (Schöttker et al., 2014), including cancer (Sluyter et al., 2021), immune system deficiency, diabetes mellitus (Zhang et al., 2020), and even cardiovascular diseases (Bolland et al., 2014; Nudy et al., 2020). However, the benefit of supplementary therapy has not been confirmed in cardiovascular diseases. Nevertheless, in a long-term randomized trial, all-cause mortality decreased if patients with low 25(OH)D serum concentrations were given vitamin D_3_ supplementation (Schöttker et al., 2014). The failure of randomized controlled trials (RCTs), to reveal the role played by vitamin D in the reduction of CVD risk, can be explained by the fact that patients with very low serum 25(OH)D concentrations are at the greatest risk. For instance, using non-linear Mendelian randomization (MR) analyses, conducted on the UK Biobank cohort, successfully supported the role of vitamin D deficiency in the risk of CVDs, which could be overlooked in standard linear MR (Zhou et al., 2022). A cohort study of 20,025 patients showed a lower risk of myocardial infarction in individuals with serum 25(OH)D concentrations ≥30 ng/mL and a lower risk of all-cause mortality with concentrations of >20 ng/mL and >30 ng/mL (Acharya et al., 2021). With respect to hypertension, in a case–control study using a community-based program of vitamin D supplementation, it was found that reaching serum 25(OH)D concentrations ≥100 nmol/L in hypertensive subjects was associated with a significant decrease in the systolic and diastolic blood pressure and mean arterial pressure (Mirhosseini et al., 2017).

Many studies have depicted the protective effect of vitamin D against various cancer types through different mechanisms like controlling tumor cell survival, differentiation, proliferation, invasiveness, and metastasis (Muñoz and Grant, 2022). A RCT on healthy postmenopausal women concluded that there was a considerable reduction of all-cancer risk through the improvement of the calcium and vitamin D nutritional status (Lappe et al., 2007).

Vitamin D was found to be effective in lowering diabetes risk in prediabetic adults, according to a recent systematic review and meta-analysis (Pittas et al., 2023). Among adults with prediabetes at a high risk for diabetes, sustained >100 nmol/L serum 25(OH)D concentrations with a daily intake of 4000 IU vitamin D_3_ considerably reduced the risk for type 2 diabetes (Dawson-Hughes et al., 2020). Insulin resistance in pregnancy could be related to vitamin D insufficiency, which is also exacerbated by excessive gestational weight gain (Rodrigues et al., 2022).

A systematic review confirmed an independent association between vitamin D deficiency and acute respiratory infections (Jolliffe et al., 2013). Vitamin D supports the antiviral and anti-inflammatory activities of airway epithelial cells (Zdrenghea et al., 2017). Showing significance for the prevention of acute respiratory tract infections, vitamin D supplementation was found safe and effective in a meta-analysis (Martineau et al., 2017) carried out before the coronavirus disease 2019 (COVID-19) pandemic. At the same time, a lack of effective treatment and vaccines at the beginning of the COVID-19 pandemic drew attention to vitamin D supplementation in order to prevent and, later, to alleviate the course of the disease (Jude et al., 2022).

A systematic review and meta-analysis concluded that vitamin D deficiency might increase the risk of COVID-19 infections and the severity of the disease but had no effect on mortality (Kaya et al., 2021). Cytokine storm and disseminated intravascular coagulation led to death in severe cases of COVID-19 infections (DiNicolantonio and O'Keefe, 2021). Vitamin D and magnesium deficiencies play a crucial role in the pathogenesis of the aforementioned conditions by reducing the cytotoxicity of NK and CD8^+^ cells that lead to increased proinflammatory death in virally infected cells and healthy cells; vitamin D is required to boost the expression of cathelicidins (DiNicolantonio and O'Keefe, 2021). A cohort retrospective study demonstrated 28% and 20% reduction in COVID-19 infections with vitamins D_2_ and D_3_, respectively, in addition to 25% and 33% reduction in COVID-19 30-day mortality, respectively (Gibbons et al., 2022). Better COVID-19 outcomes were associated with achieving serum 25(OH)D concentrations ≥30 ng/mL using either cholecalciferol or calcifediol in a population-based cohort study (Oristrell et al., 2022). According to a pilot randomized clinical study, high doses of calcifediol significantly reduced ICU admissions among COVID-19 hospitalized patients (Entrenas Castillo et al., 2020).

The purpose of this study was to examine the basic characteristics of patients with a known vitamin D status in order to identify the rates of vitamin D deficiency and its relation to certain diseases. The authors also made an attempt to assess the seasonality and trends over time, and to reveal the indirect impact of the COVID-19 pandemic on vitamin D supplementation through population-level vitamin D consumption.

## Methods

### The study population

All the test results of serum 25(OH)D concentrations as anonymized data were obtained, prospectively, from the clinical data registry of a tertiary teaching hospital covering the period between 1 January 2015 and 30 June 2021. The catchment population was approximately 700,000 inhabitants. In the catchment area, patients had been referred to one of the outpatient units or were hospitalized in an inpatient department, or their blood samples had been sent in for the determination of serum 25(OH)D concentrations at a regional reference laboratory. In addition to the values measured for the first and subsequent times of the vitamin D status in every patient, the subjects’ age, gender, time of sampling, ward, and diagnoses were also entered in the registry in order to create a database and analyze the data on all these patients.

### Laboratory examination

The automated LIAISON DiaSorin total 25 OH vitamin D chemiluminescence immunoassay (CLIA) (DiaSorin Inc., Stillwater, MN, USA) was used to analyze serum 25(OH)D concentrations. The inter-assay CV was <7.8% for 25(OH)D (lower detection limit: 10 nmol/L; upper detection limit: 375 nmol/L).

Our laboratory participates in the DEQAS EQA scheme and is compliant with the set standards.

### Drug utilization research

Based on the ATC/DDD (Anatomical Therapeutic Chemical classification of medicines/defined daily dose) system of the World Health Organization (WHO), vitamin D consumption (pharmacy sale) data were retrieved from the database of the National Health Insurance Fund of Hungary in order to calculate the DDD/1,000 inhabitants/day (DID) in the region investigated as the gold standard of drug consumption according to the WHO (WHO, 2013; WHOCC, 2016).

### Statistical analysis

Descriptive statistics and odds ratios with their 95% confidence intervals [OR (95% CI)] were calculated. The two-sample *t*-test and F-test were used to analyze our patients’ data. Significant differences were considered if *p* <0.05.

Statistical analysis was carried out using SPSS for Windows 22.0 (IBM, USA) and Microsoft Office Excel 2016.

Ethical approval was obtained from the Regional and Institutional Ethics Committee (RKEB 5865-2021).

## Results

In the period covered, serum 25(OH)D concentrations were investigated in 45,567 samples. Of them, 18,631 patients had a single measurement and 8,043 (30.2%) patients had repeated measurements. They had altogether 26,936 findings (Table 1).

**TABLE 1:** Number of patients and the number of subsequent 25(OH)D tests.

Number of patients	Number of subsequent findings	Common diagnoses
3,916	2	Sine morbo, hypertension, CKD, Raynaud’s sy., osteoporosis, RA, infertility, M. Crohn, and hypothyroidism
1,736	3	CKD, sine morbo, hypertension, RA, Raynaud’s sy., colitis ulcerosa, M. Crohn, hypothyroidism, osteoporosis, and SM
950	4	CKD, sine morbo, hypertension, osteoporosis, M. Crohn, Raynaud’s sy., hypothyroidism, and SM
485	5	CKD, sine morbo, hypertension, SM, hypertension, M. Crohn, osteoporosis, and hypothyroidism
310	6	CKD, sine morbo, hypertension, SM, hypothyroidism, M. Crohn, colitis ulcerosa, osteoporosis, and RA
202	7	SM, hypertension, CKD, CVDs, thyroiditis, hypothyroidism, osteoporosis, M. Crohn, colitis ulcerosa, and sine morbo
141	8	SM, hypertension, CKD, CVDs, thyroiditis, hypothyroidism, osteoporosis, vitamin D deficiency, and M. Crohn
89	9	SM, CKD, CVDs, thyroiditis, hypothyroidism, dermatitis, hypertension, M. Gaucher, and M. Anderson–Fabry
75	10	SM, hypertension, CKD, hypothyroidism, thyroiditis, myasthenia gravis, and CVDs
58	11	SM, hypertension, CKD, RA, and CVDs
38	12	SM, hypertension, thyroiditis, CKD, and ITP
18	13	SM, hypertension, M. Gaucher, lung cancer, and BPH
10	14	M. Recklinghausen, M. Gaucher, CVDs, osteoporosis, lung cancer, hypertension, thyroiditis, CKD, and BPH
4	15	Lung cancer, CVDs, and CML
7	16	CVDs, osteoporosis, hypothyroidism, ITP, and thyroiditis
2	17	Hypertension and juvenile dermatomyositis
1	18	Hypertension
1	26	Lung cancer

CKD, chronic kidney disease; CVD, cardiovascular disease; RA, rheumatoid arthritis; SM, sclerosis multiplex; ITP, immune thrombocytopenic purpura; M, morbus; sy., syndrome; BPH, benign prostatic hyperplasia; CML, chronic myeloid leukemia.

**FIGURE 1:**
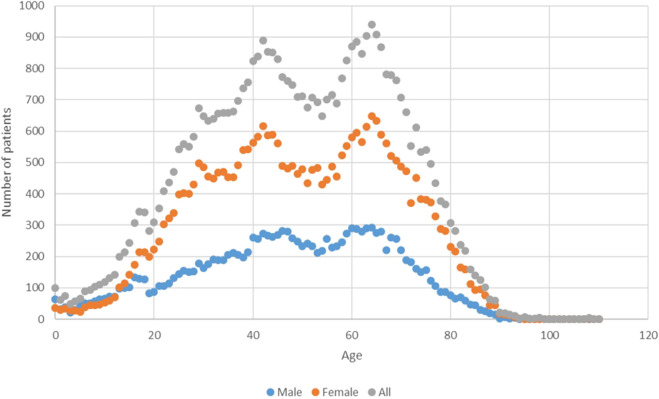
Patients’ distribution by age.

The mean age was 49 ± 19.1 years. The age distribution showed two distinct peaks (Figure 1). The number of male and female patients was 14,401 (31.6%) and 31,166 (68.4%), respectively. Their mean ages were 47.87 ± 19.6 years and 49.53 ± 18.8 years, respectively (*p* <0.0001).

**FIGURE 2:**
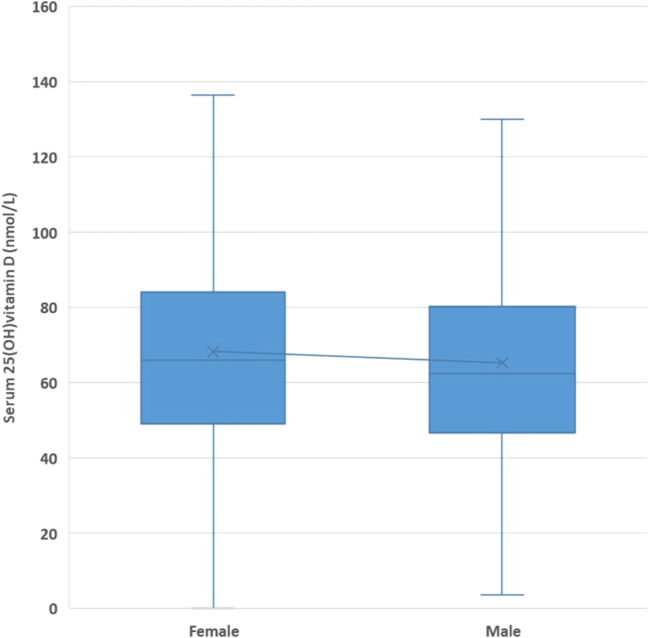
Mean serum 25(OH)D concentrations by gender.

The mean serum 25(OH)D concentration was 67.5 ± 28.4 nmol/L (male patients: 65.4 ± 28.2 nmol/L and female patients: 68.4 ± 28.4 nmol/L; *p* <0.0001) (Figure 2).

Outpatients had significantly higher mean serum 25(OH)D concentrations than inpatients, which is 67.6 ± 28.4 nmol/L *vs.* 64.3 ± 36.1 nmol/L, respectively (*p* <0.0001).

**FIGURE 3:**
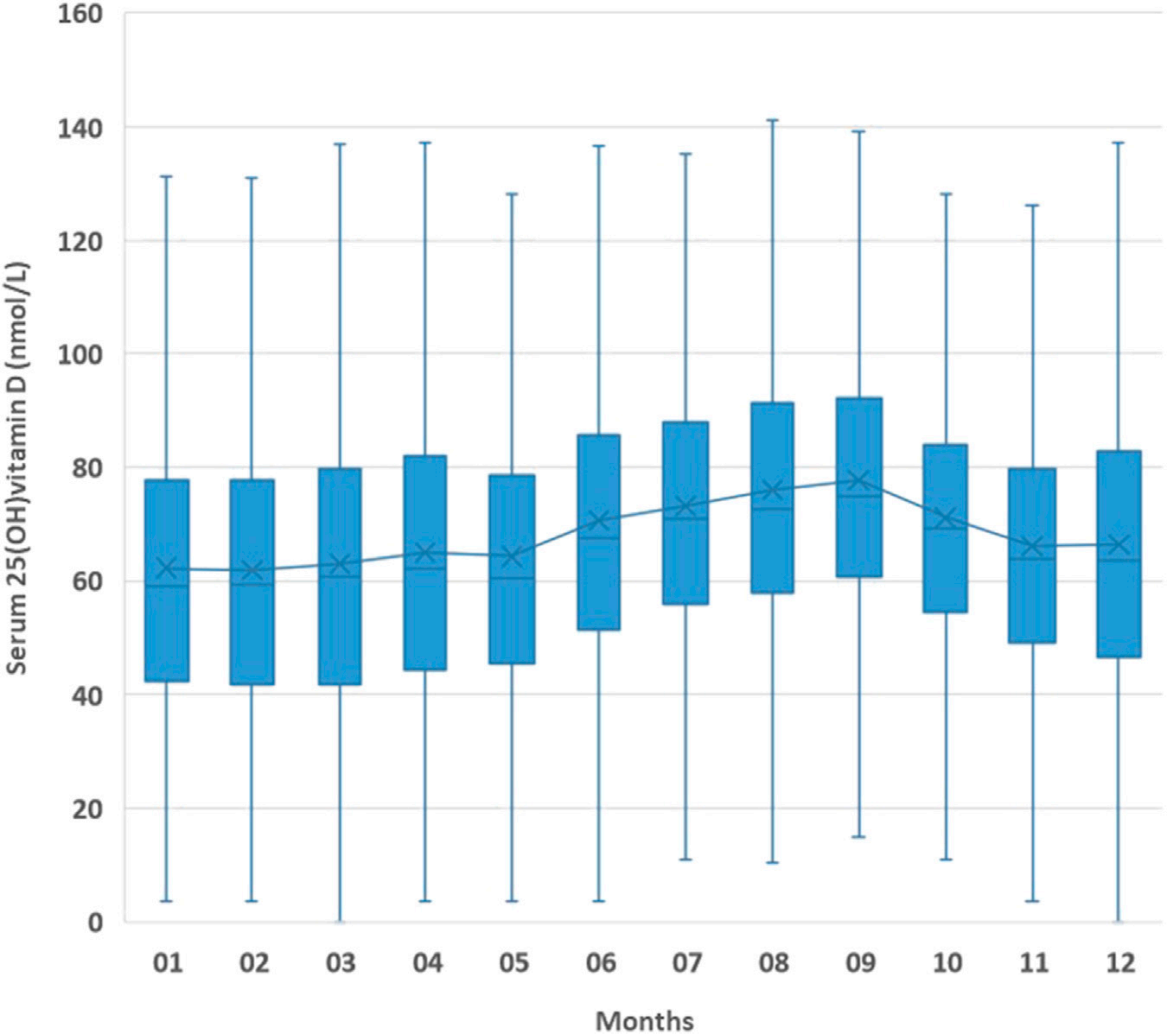
Mean serum 25(OH)D concentrations by months.

**FIGURE 4:**
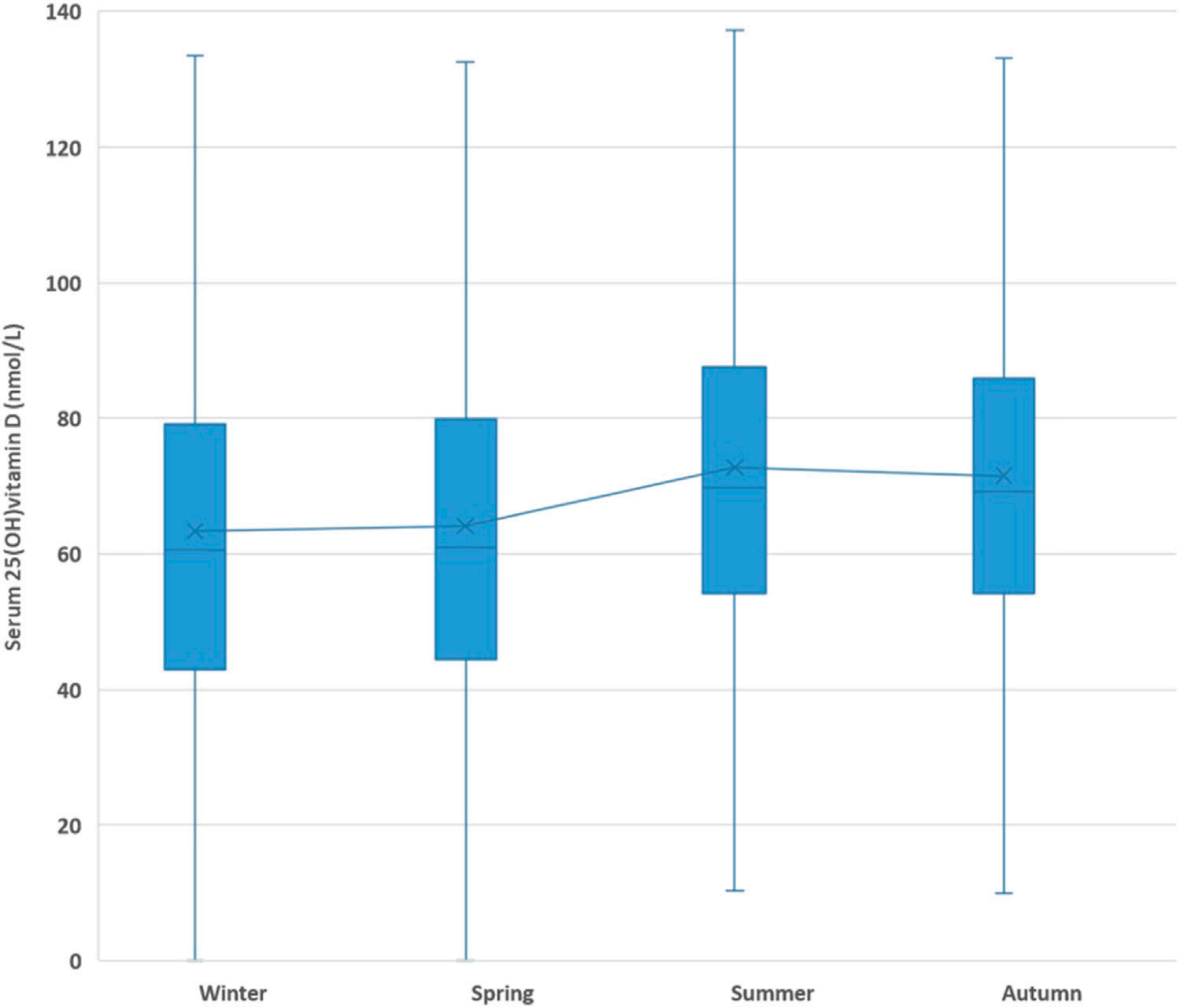
Seasonality of the serum 25(OH)D concentration.

The mean serum 25(OH)D concentration had changed during the year and showed a peak in September and a minimum in February (Figures 3, 4).

**FIGURE 5:**
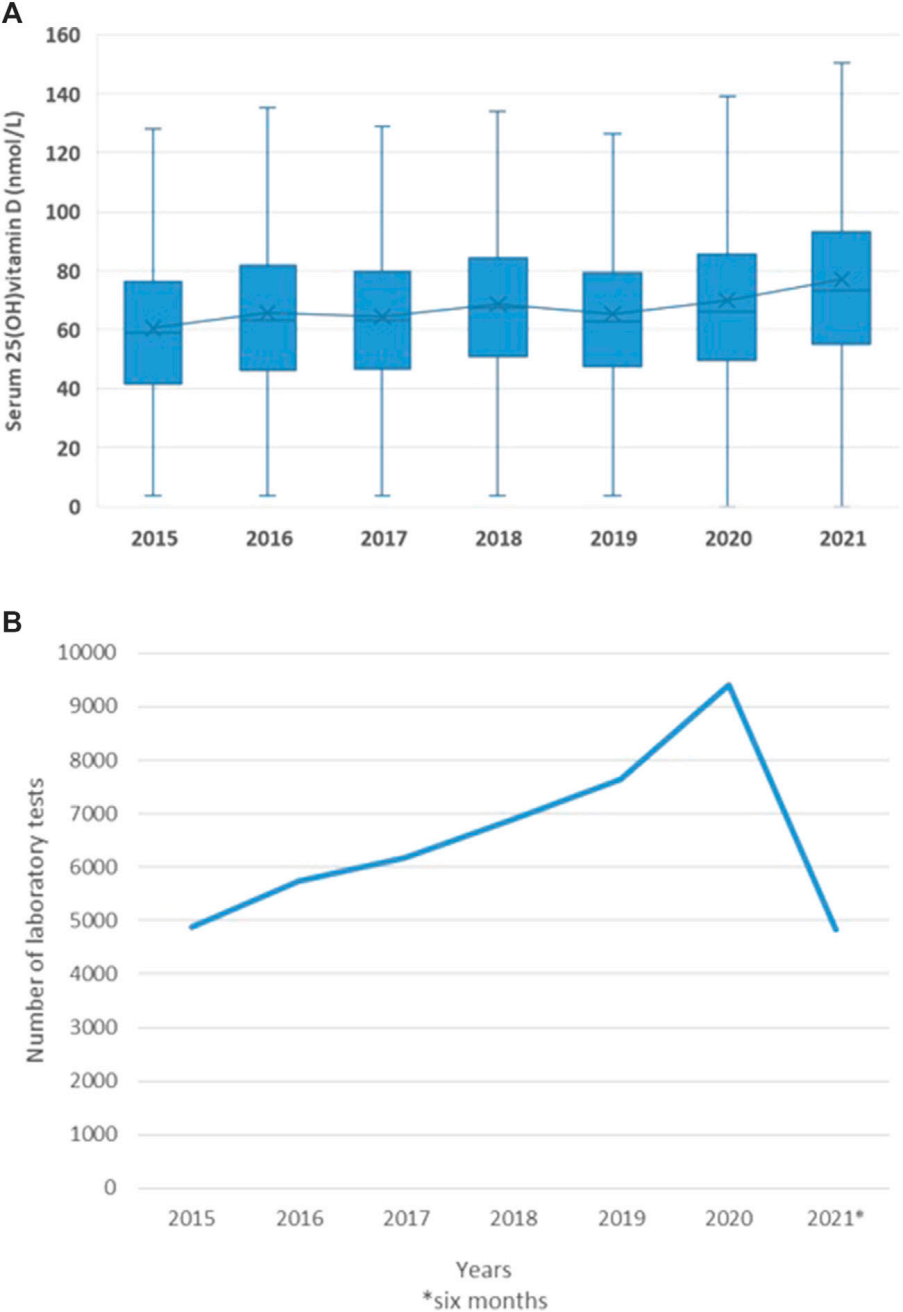
Mean serum 25(OH)D concentration by years **(A)** and the number of laboratory tests **(B)**.

**FIGURE 6:**
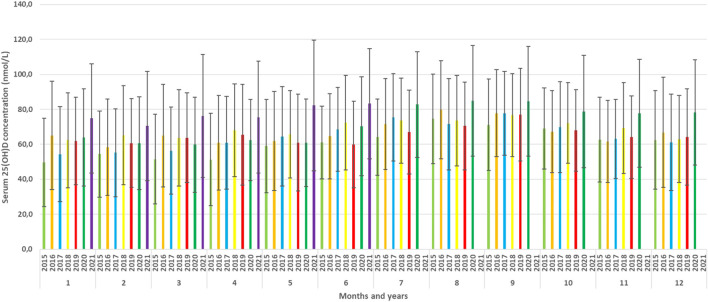
Comparison of the monthly mean serum 25(OH)D concentration by years.

**FIGURE 7:**
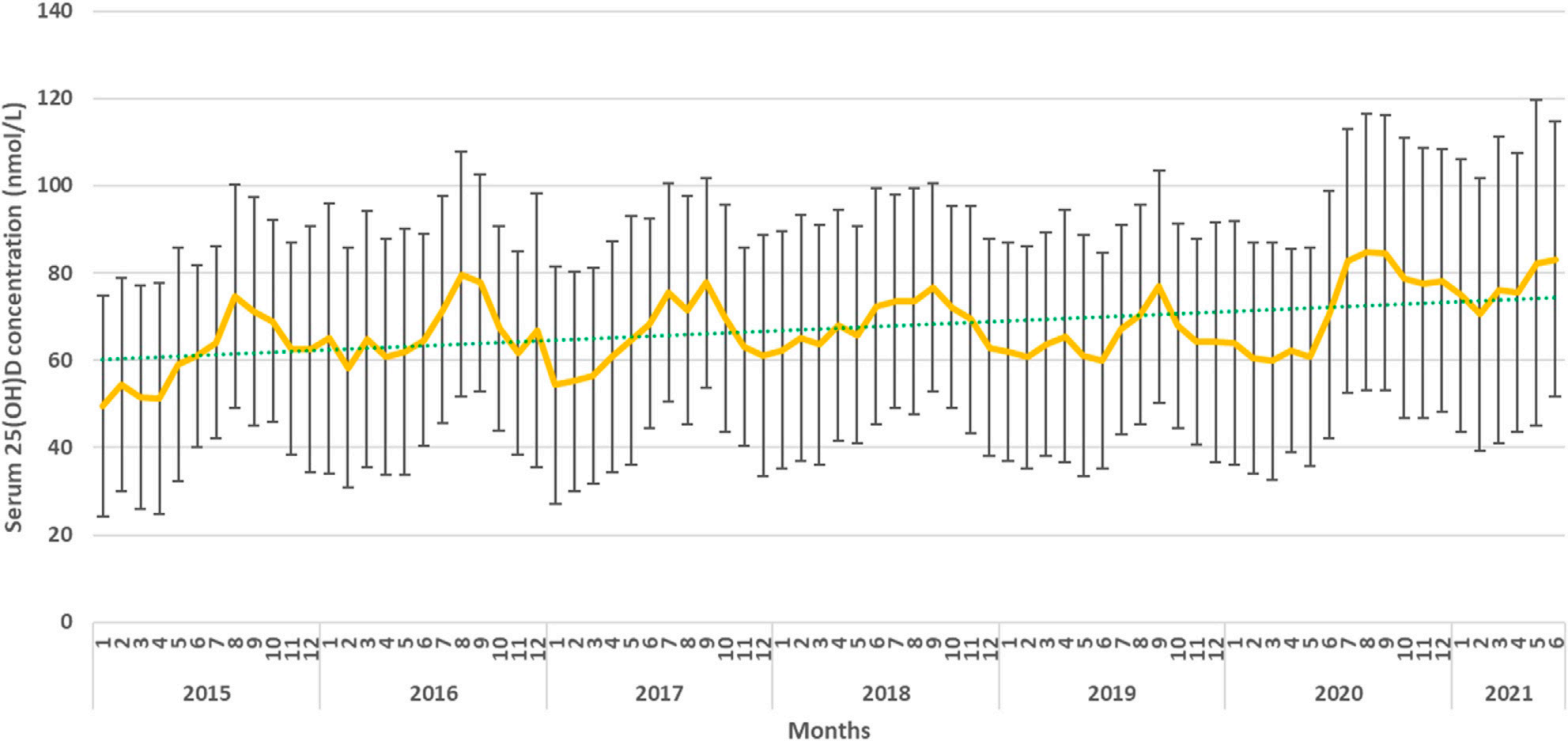
Trend of mean serum 25(OH)D concentrations.

The number of laboratory tests increased over the years. Mean serum 25(OH)D concentrations showed an increasing trend over time and a remarkable increase in 2021 (Figures 5–7).

Nearly 70% of patients’ 25(OH)D values were in the reference range. Twenty percent of all patients had hypovitaminosis D, and just over 7% of patients had vitamin D deficiency. Male subjects had higher odds for hypovitaminosis D and vitamin D deficiency than female subjects (Table 2). Vitamin D toxicity was a very rare event (altogether two patients, 0.004%). Male subjects also had higher odds for having lower serum 25(OH)D concentrations using the 75 nmol/L cut-off value (Table 2).

**TABLE 2:** Proportion of vitamin D deficiency.

Vitamin D status		Number of patients (%)		Male subjects (%)		Female subjects (%)		OR (95% CI)			*p*-value
Severe vitamin D deficiency (<4 nmol/L)		92 (0.2)		27 (0.19)		65 (0.19)		0.94 (0.49–.1.39)			0.88
Vitamin D deficiency (5–29 nmol/L)		3,122 (6.85)		1,062 (7.37)		2,060 (6.61)		**1.17 (1.09–1.25)**			**0.00007**
Hypovitaminosis (30–49 nmol/L)		9,195 (20.18)		3,213 (22.31)		5,982 (19.19)		**1.22 (1.17–1.27)**			**<0.00001**
Reference range (50–125 nmol/L)		31,644 (69.45)		9,673 (67.17)		21,971 (70.5)		*ref.*			
High (>125 nmol/L)		1,512 (3.32)		425 (2.95)		1,087 (3.49)		0.89 (0.77–1.00)			**0.045**
Toxicity (>500 nmol/L)		2 (0.004)		1 (0.007)		1 (0.003)		2.27 (0–5.04)			0.86
All		45,567 (100)		14,401 (100)		31,166 (100)		-			-

75 nmol/L cutoff		Number of patients (%)		Male subjects (%)		Female subjects (%)		OR (95% CI)			*p*-value
<75 nmol/L		29,687 (65.1)		9,872 (21.7)		19,815 (43.5)		**1.25 (1.21-1.29)**			**<0.00001**
≥75 nmol/L		15,880 (34.8)		4,529 (9.9)		11,351 (24.9)		**-**			**-**

Statistically significant values are highlighted in bold.

As for inpatients, there were significantly more of them who had certain infectious and parasitic diseases, neoplasms, mental and behavioral disorders, diseases of the circulatory system or respiratory system, and certain conditions originating in the perinatal period (*p* <0.0001), while diseases of the nervous system, eye and adnexa, digestive system, musculoskeletal system and connective tissue, genitourinary system, and “sine morbo” occurred significantly more often among outpatients than in inpatients (*p* <0.0001).

Vitamin D deficiency and hypovitaminosis D were between 16.5% and 47.8% in all disease classifications used [chapters of the International Statistical Classification of Diseases and Related Health Problems (ICD-10)] (Table 3).

**TABLE 3:** Number and mean serum 25(OH)D concentrations of patients by chapters of the International Statistical Classification of Diseases and Related Health Problems.

ICD-10 chapter (code)	Serum 25(OH)D concentration (nmol/L)			
	<30	30–49	50–125	>125
	Number of patients (mean serum 25(OH)D concentration—nmol/L) [OR(95% CI)]			
Certain infectious and parasitic diseases (A00–B99)	17 (3.66) [0.67(0.18–1.15)]	94 (20.22) [0.9(0.68–1.13)]	333 (71.61) [1.11(0.9–1.31)]	**21 (4.52)** [1.38(0.94–1.82)]
Neoplasms (C00–D48)	**60 (5.11)** [0.95(0.69–1.21)]	213 (18.14) [0.79(0.64–0.94)]*	832 (70.87) [1.07(0.94–1.2)	**69 (5.88)** [1.86(1.6–2.1)]*
Diseases of the blood and blood-forming organs and certain disorders involving the immune mechanism (D50–D89)	**47 (5.04)** [0.94(0.64–1.23)]	180 (19.29) [0.85(0.69–1.02)]	674 (72.24) [1.15(1.0–1.29)]	32 (3.43) [1.03(0.68–1.39)]
Endocrine, nutritional, and metabolic diseases (E00–E90)	224 (4.44) [0.8(0.66–0.94)]*	1,006 (19.94) [0.88(0.8–0.95)]*	3,656 (72.47) [1.18(1.11–1.24)]*	159 (3.15) [0.94(0.77–1.11)]
Mental and behavioral disorders (F00–F99)	**8 (9.09)** [1.77(1.04–2.5)]*	18 (20.45) [0.92(0.4–1.44)]	57 (64.77) [0.81(0.37–1.25)]	**5 (5.68)** [1.76(0.85–2.66)]
Diseases of the nervous system (G00–G99)	118 (4.31) [0.79(0.6–0.98)]*	473 (17.29) [0.73(0.63–0.84)]*	2025 (74.04) [1.27(1.18–1.36)]*	**119 (4.35)** [1.35(1.16–1.54)]*
Diseases of the eye and adnexa (H00–H59)	**6 (5.31)** [0.99(0.17–1.81)]	16 (14.16) [0.59(0.06–1.12)]	85 (75.22) [1.34(0.91–1.76)]	**6 (5.31)** [1.63(0.81–2.46)]
Diseases of the ear and mastoid process (H60–H95)	2 (2.90) [0.53(0–1.93)]	**15 (21.74)** [0.99(0.42–1.56)]	50 (72.46) [1.16(0.63–1.69)]	2 (2.90) [0.87(0–2.28)]
Diseases of the circulatory system (I00–I99)	**362 (6.25)** [1.21(1.09–1.32)]*	**1,349 (23.28)** [1.1(1.03–1.16)]*	3,921 (67.67) [0.91(0.85–0.97)]*	162 (2.80) [0.82(0.65–0.98)]*
Diseases of the respiratory system (J00–J99)	31 (2.99) [0.54(0.18–0.9)]*	**237 (22.88)** [1.06(0.91–1.21)]	738 (71.24) [1.09(0.96–1.23)]	30 (2.90) [0.87(0.5–1.23)]
Diseases of the digestive system (K00–K93)	148 (4.71) [0.86(0.69–1.03)]	616 (19.59) [0.86(0.77–0.95)]*	2,286 (72.69) [1.18(1.1–1.27)]	95 (3.02) [0.9(0.69–1.11)]
Diseases of the skin and subcutaneous tissue (L00–L99)	**39 (5.95)** [1.12(0.79–1.45)]	**148 (22.60)** [1.04(0.86–1.23)]	451 (68.85) [0.97(0.81–1.14)]	17 (2.60) [0.77(0.29–1.26)]
Diseases of the musculoskeletal system and connective tissue (M00–M99)	**280 (5.35)** [1.0(0.87–1.13)]	1,049 (20.03) [0.88(0.81–0.95)]*	3,722 (71.08) [1.09(1.03–1.16)]*	185 (3.53) [1.07(0.92–1.23)]
Diseases of the genitourinary system (N00–N99)	**670 (9.08)** [2.05(1.96–2.15)]*	**1,783 (24.17)** [1.17(1.11–1.23)]*	4,718 (63.96) [0.74(0.69–0.79)]*	206 (2.79) [0.81(0.66–0.96)]*
Pregnancy, childbirth, and the puerperium (O00–O99)	**4 (5.26)** [0.98(0–1.99)]	11 (14.47) [0.6(0–1.24)]	60 (78.95) [1.65(1.1–2.2)]	1 (1.32) [0.39(0–2.36)]
Certain conditions originating in the perinatal period (P00–P96)	**3 (6.52)** [1.23(0.06–2.4)]	**19 (41.30)** [2.52(1.93–3.1)]*	18 (39.13) [0.28(0–0.87)]	**6 (13.04)** [4.38(3.52–5.24)]*
Congenital malformations, deformations, and chromosomal abnormalities (Q00–Q99)	5 (2.99) [0.54(0–1.44)]	29 (17.37) [0.75(0.35–1.15)]	124 (74.25) [1.27(0.92–1.62)]	**9 (5.39)** [1.66(0.99–2.34)]
Symptoms, signs, and abnormal clinical and laboratory findings, not classified elsewhere (R00–R99)	**181 (4.90)** [0.9(0.75–1.06)]	**832 (22.54)** [1.04(0.96–1.12)]	2,562 (69.39) [1.0(0.92–1.07)]	117 (3.17) [0.95(0.76–1.14)]
Injury, poisoning, and certain other consequences of external causes (S00–T98)	1 (1.27) [0.23(0–2.2)	12 (15.19) [0.64(0.02–1.25)]	64 (81.01) [1.88(1.32–2.44)]*	2 (2.53) [0.76(0–2.16)]
Codes for special purposes (U00–U85)	0 (0.00) [N/C]	**6 (33.33)** [1.79(0.81–2.77)]	11 (61.11) [0.69(0–1.64)	**1 (5.56)** [1.71(0–3.73)]
Sine morbo (U9990)	115 (1.96) [0.32(0.13–0.51)]	**1,549 (26.35)** [1.33(1.27–1.39)]*	4,023 (68.44) [0.95(0.89–1.01)]	191 (3.25) [0.97(0.82–1.13)]
Unknown	**121 (6.93)** [1.33(1.14–1.52)]*	312 (17.87) [0.77(0.65–0.89)]*	1,234 (70.68) [1.06(0.96–1.17)]	**79 (4.52)** [1.4(1.17–1.63)]*

Bold: above the mean; *, statistically significant; N/C, not computable.

Patients with diseases of the circulatory system (OR: 1.14; 1.08–1.20; *p* <0.00001), genitourinary system (OR: 1.41; 1.36–1.47; *p* <0.00001), certain conditions originating in the perinatal period (OR: 2.45; 1.87–3.03; *p* <0.00001), and sine morbo (OR: 1.06; 1.01–1.12; *p* <0.00001) had statistically higher odds for vitamin D deficiency.

Twenty ICD-10 blocks had significantly higher odds for vitamin D deficiency and hypovitaminosis D, including *in situ* neoplasms (D00–D09), diabetes mellitus (E10–E14), obesity (E65–E68), and organic—including symptomatic mental disorders (F00–F09), epilepsy (G40–G41), cardiovascular diseases (I10–I15, I30–I52, and I80–I89), non-infective enteritis and colitis (K50–K52), papulosquamous disorders (L40–L45), renal failure (N17–N19), and certain conditions originating in the perinatal period (P20–P29 and P35–P39) (Table 4).

**TABLE 4:** Statistically significant high odds for vitamin D deficiency by blocks of the International Statistical Classification of Diseases and Related Health Problems.

ICD-10 block (code)	Number of patients with serum 25(OH)D concentrations <50 nmol/L	Odds ratio (95% confidence interval)	*p*-value
*In situ* carcinoma (D00–D09)	6	2.67 (1.07–4.27)	0.001
Diabetes mellitus (E10–E14)	252	1.36 (1.21–1.52)	<0.00001
Obesity and other hyperalimentation (E65–E68)	73	1.81 (1.51–2.11)	<0.00001
Organic, including symptomatic mental disorders (F00–F09)	7	2.67 (1.63–3.72)	<0.00001
Epilepsy and status epilepticus (G40–G41)	23	2.37 (1.80–2.93)	<0.00001
Hypertensive diseases (I10–I15)	1,072	1.16 (1.08–1.23)	<0.00001
Other forms of heart diseases (I30–I52)	115	1.34 (1.11–1.56)	<0.00001
Diseases of veins, lymphatic vessels, and lymph nodes, not classified elsewhere (I80–I89)	70	1.88 (1.57–2.18)	<0.00001
Non-infective enteritis and colitis (K50–K52)	312	1.19 (1.05–1.32)	<0.00001
Papulosquamous disorders (L40–L45)	47	1.77 (1.40–2.14)	<0.00001
Glomerular diseases (N00–N08)	48	1.63 (1.27–1.99)	<0.00001
Renal failure (N17–N19)	1,951	1.75 (1.69–1.81)	<0.00001
Respiratory and cardiovascular disorders specific to the perinatal period (P20–P29)	7	6.24 (4.89–7.59)	<0.00001
Infections specific to the perinatal period (P35–P39)	8	2.67 (1.69–3.65)	<0.00001
Symptoms and signs involving the urinary system (R30–R39)	11	1.84 (1.07–2.61)	<0.00001
Abnormal findings on the examination of urine, without diagnosis (R80–R82)	11	2.10 (1.31–2.89)	<0.00001
Sine morbo (U9990)	1,664	1.06 (1.01–1.12)	<0.00001

**FIGURE 8:**
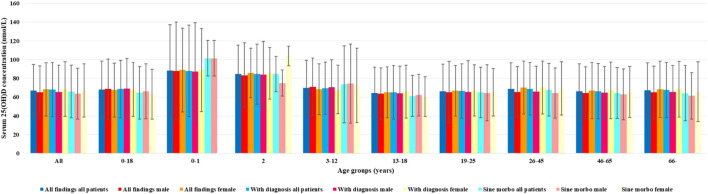
Findings of patients’ mean serum 25(OH)D concentrations by diagnostic groups.

Patients with sine morbo diagnoses had statistically significant lower serum 25(OH)D concentrations in all patients and among male and female subjects; by age stratification, we had the following subgroups: 0–18, 13–18, 46–65, and above 66 years (Table 5; Figure 8).

**TABLE 5:** Comparison of patients’ mean serum 25(OH)D concentrations with healthy individuals’ findings by age and sex.

	All findings		Patients with diagnosis		Sine morbo diagnosed patients		*p*-value	
Age (year), *sex*	Number of patients	Mean ± SD (nmol/L)	Number of patients	Mean ± SD (nmol/L)	Number of patients	Mean ± SD (nmol/L)	Dg *vs.* all	Dg *vs.* Sm
All	45,567	67 ± 28	39,690	68 ± 29	5,877	66 ± 28	0.31	**<0.0001**
M	*14,401*	*65.3 ± 28.2*	*12,186*	*65.6 ± 28.6*	*2,215*	*63.7 ± 27.1*	0.39	**0.004**
F	*31,166*	*68.3 ± 28.4*	*27,504*	*68.5 ± 29.2*	*3,662*	*67.1 ± 28.4*	0.4	**0.007**
0–18	2,844	68.1 ± 30.3	2,474	68.6 ± 30.6	370	64.7 ± 28.1	0.55	**0.02**
M	*1,351*	*68.6 ± 32.1*	*1,158*	*69.0 ± 32.5*	*193*	*66.1 ± 29.3*	0.76	0.24
F	*1,493*	*67.6 ± 28.7*	*1,316*	*68.2 ± 28.9*	*177*	*63.2 ± 26.7*	0.58	**0.03**
0–1	99	88.3 ± 49	96	87.9 ± 48.8	3	101.4 ± 19	0.95	0.63
M	*63*	*88.0 ± 52*	*60*	*87.3 ± 52*	*3*	*101.4 ± 19*	0.94	0.64
F	*36*	*88.9 ± 44.6*	*36*	*88.9 ± 44.3*	*0*	*0*	1.0	N/C
2	136	84.6 ± 30.8	130	84.6 ± 32	6	84.7 ± 18.8	1.00	0.99
*M*	*71*	*83.3 ± 34.6*	*67*	*83.8 ± 35.8*	*4*	*75.1 ± 14.1*	0.93	0.63
F	*65*	*85.9 ± 26.4*	*63*	*85.4 ± 27.6*	*2*	*104.0 ± 10.5*	0.92	0.34
3–12	961	69.8 ± 29.3	880	69.4 ± 27.8	81	73.7 ± 41.2	0.76	0.2
M	*527*	*70.8 ± 31*	*478*	*70.5 ± 29.5*	*49*	*74.4 ± 42.2*	0.88	0.4
F	*434*	*68.5 ± 27.2*	*402*	*68.2 ± 25.8*	*32*	*72.7 ± 39.6*	0.87	0.37
13–18	1,648	64.5 ± 27.4	1,368	65.1 ± 28.5	280	61.3 ± 22	0.65	**0.04**
M	*690*	*63.6 ± 27.7*	*553*	*64.0 ± 29.1*	*137*	*62.1 ± 22.1*	0.8	0.48
F	*958*	*65.1 ± 27.2*	*815*	*65.9 ± 28.1*	*143*	*60.5 ± 21.2*	0.54	**0.03**
19–25	2,802	66.4 ± 28.7	2,339	66.7 ± 29	463	65.0 ± 27	0.71	0.25
M	*771*	*65.3 ± 32.7*	*607*	*65.5 ± 33.4*	*164*	*64.5 ± 29.9*	0.91	0.72
F	*2031*	*66.8 ± 27*	*1732*	*67.1 ± 27.3*	*299*	*65.3 ± 25.3*	0.29	0.74
26–45	14,195	68.8 ± 28	11,866	68.9 ± 28	2,329	67.8 ± 28	0.77	0.083
M	*4,164*	*65.6 ± 27.1*	*3,400*	*65.8 ± 27.2*	*764*	*64.5 ± 26.8*	0.75	0.23
F	*10,031*	*70.1 ± 28.2*	*8,466*	*70.2 ± 28.2*	*1,565*	*69.4 ± 28.4*	0.81	0.3
46–65	15,475	66.2 ± 29.2	13,621	66.4 ± 29.5	1,854	64.4 ± 27.2	0.56	**0.006**
M	*5,157*	*64.5 ± 27.7*	*4,388*	*64.8 ± 27.8*	*769*	*62.9 ± 27.1*	0.6	0.08
F	*10,318*	*67.0 ± 29.9*	*9,233*	*67.2 ± 30.3*	*1,085*	*65.5 ± 27.2*	0.64	0.08
66-	10,251	67.4 ± 29.4	9,390	67.7 ± 29.3	861	64.2 ± 29.4	0.47	**0.0008**
M	*2,958*	*65.0 ± 28*	*2,633*	*65.4 ± 28.4*	*325*	*61.5 ± 24.8*	0.6	**0.02**
F	*7,293*	*68.3 ± 30*	*6,757*	*68.5 ± 29.6*	*536*	*65.9 ± 31.8*	0.69	0.05

M, male individual; F, female individual; SD, standard deviation; Dg, patients with diagnosis; Sm, sine morbo.

Statistically significant values are highlighted in bold.

**FIGURE 9:**
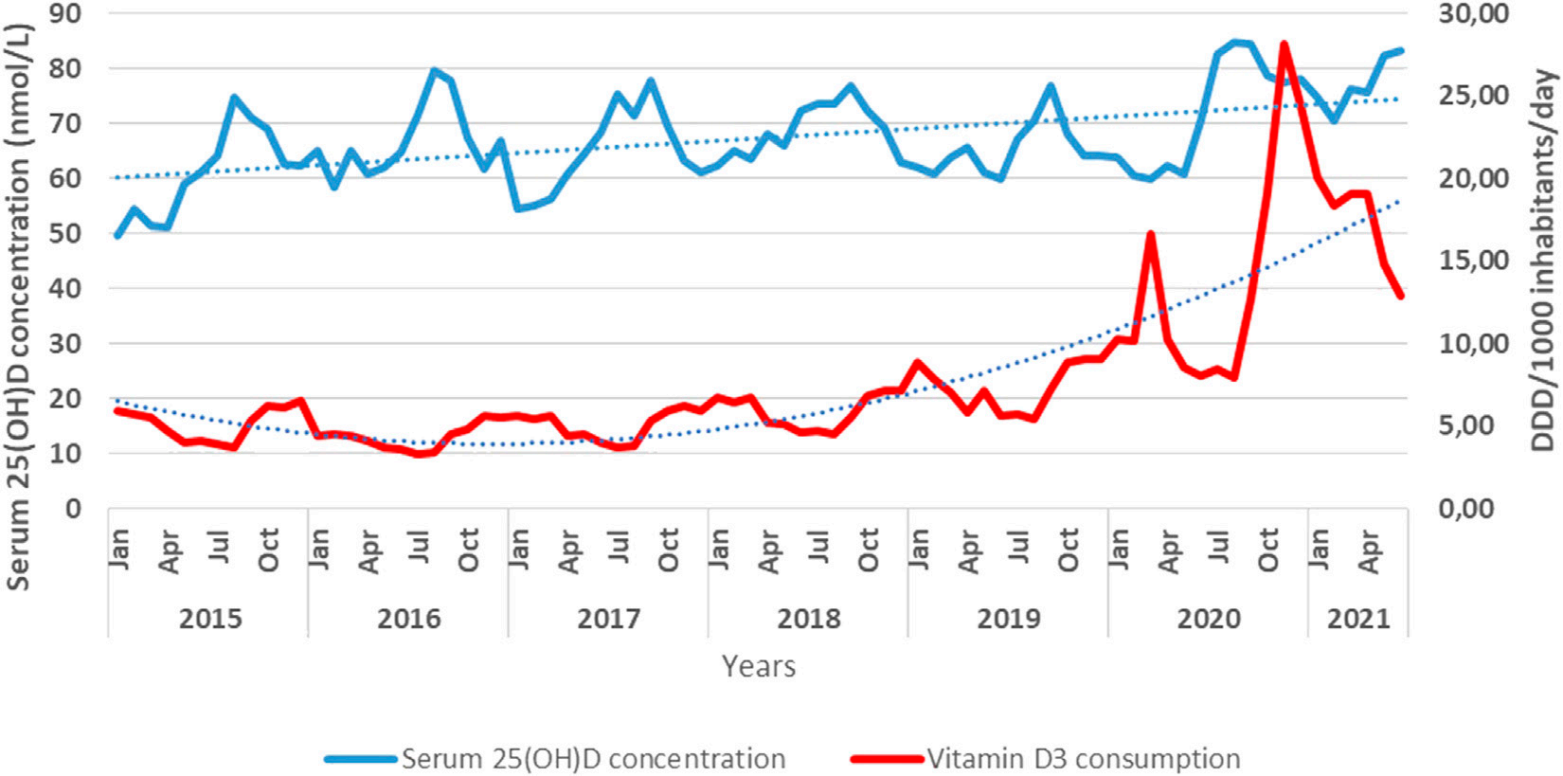
Monthly vitamin D consumption by DDD/1,000 inhabitants/year and mean serum 25(OH)D concentrations.

**FIGURE 10:**
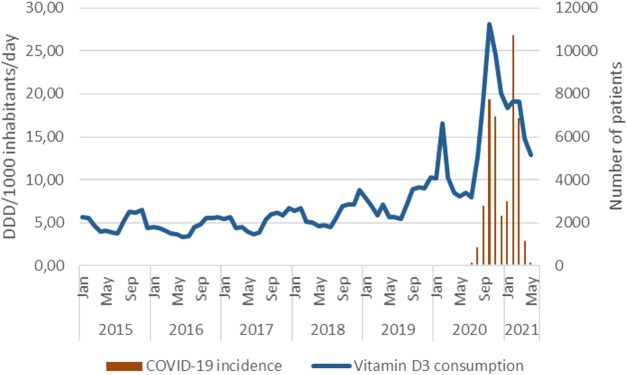
Monthly vitamin D_3_ consumption by DDD/1,000 inhabitants/year and COVID-19 incidence.

Vitamin D_3_ consumption showed seasonality, being higher in autumn and winter. A slight increase started in the season of 2017/18 (Figure 9). Two huge peaks characterized the vitamin D_3_ consumption at the beginning of 2020 and 2021 related to COVID-19 waves (Figure 10).

## Discussion

Nowadays, the importance of vitamin D is becoming better recognized, even among non-medical people and by the media. Although the biochemical role of this vitamin is not always clarified, clinical practice draws attention to its significance. We have been the first in our region to examine its role from a pharmacoepidemiological aspect in a study of a large sample (*n* = 45,567). We think it is important because Hungary has been characterized by a low intake of vitamin D_3_, as reported in a recent dietary survey (Cashman, 2022).

During the study period, almost one-third of the patients had repeated measurements, suggesting that healthcare professionals and their patients considered that the follow-up of serum 25(OH)D concentration was important.

The seasonal fluctuation of serum 25(OH)D concentrations has been known for a long time, and it is public knowledge that these concentrations are lowest in the winter (Schöttker et al., 2014). Providing confirmation on this, our study showed that serum 25(OH)D concentrations reached a peak in September and reached a nadir in February. Altogether, serum 25(OH)D concentrations increased gradually over time, but seasonal fluctuations could be detected each year. Skeletal muscle cells play an important role as a functional store of accumulated 25(OH)D during winter, and it is proposed that the decreased muscle function due to a lack of exercise or malnutrition may affect this (Mason et al., 2019). A possible mechanism for this is the uptake of the vitamin D-binding protein (DBP) from the blood into the muscle cell cytoplasm where it binds to cytoplasmic actin and diffuses back into the blood as the intracellular DBP undergoes proteolytic breakdown, a cycling process in and out of the muscle that seems to be upregulated in winter to cope with the decreased serum 25(OH)D concentration (Mason et al., 2019).

Maintained >100 nmol/L serum 25(OH)D concentrations may reduce the higher incidence of type 2 diabetes, particularly among prediabetic patients with a high risk for diabetes, at the end of spring (Dawson-Hughes et al., 2020). A study found that more than 80% of their winter cholecalciferol needs of heathy male individuals were obtained from the preceding summer months through cutaneously synthesized D_3_ accumulations in response to solar radiation (Heaney et al., 2003). In our region, after August, the mean solar radiation energy declines and is the lowest in December (680 MJ/m^2^ and 80 MJ/m^2^, respectively); this decrement is logarithmic, and there is a significant reduction between September and October. To avoid the emptying of vitamin D depots and considering the 8 weeks required to adapt, patients and the general population need to start supplementation as early as September. In accordance with the aforementioned information and that based on our findings, vitamin D_3_ supplementation should be started in September in order to prevent the winter decline starting through October.

Similarly, we found seasonal fluctuations of the consumption of vitamin D_3_ products in this study. The number of laboratory tests increased parallel to the growing public awareness of the importance of the vitamin D status, reflecting the population’s health consciousness. Vitamin D_3_ consumption in our country had also risen slightly by the end of 2019 due to health education highlighting the importance of vitamin D supplementation. Nevertheless, it must be kept in mind that supplementation is a complex issue since not only health conscious attitudes but economic problems can also play a role in it. Furthermore, an individual may get vitamin D from a wide array of sources including solar radiation, food (either fortified or not), dietary supplements, and over-the-counter and prescription-only medicines. Our data have been based on prescription-only preparation data that are openly available and reliable. In both our previous study and a recent pilot study on retail pharmacies, we observed a similar increase in the consumption of OTC vitamin D_3_ in terms of its dynamics and magnitude.

Similar to the results published by Jude et al., vitamin D_3_ supplementation was used in the treatment and prevention of infections caused by COVID-19 (Jude et al., 2022). In autumn and winter, the pre-COVID-19 pandemic was characterized by a 1.5–2-fold increase in the consumption of vitamin D_3_ products. Highlighting the impact of education by healthcare professionals and through media on the population, an exponential increase in the consumption of vitamin D_3_ products was seen twice during the COVID-19 pandemic, coinciding with COVID-19 pandemic waves. Kaufman et al. found lower serum 25(OH)D concentrations in association with higher COVID-19 positivity (Kaufman et al., 2020). It seems that the consumption of vitamin D_3_ remained 2–3 times higher than that in the pre-COVID-19 pandemic era.

Although female individuals were significantly overrepresented in this study, the distribution by age did not differ. This might show that healthcare professionals are aware of the fact that female individuals need to get their serum 25(OH)D concentrations checked regularly, e.g., due to osteoporosis, and that they are more often given vitamin D supplementation. Our data show higher serum 25(OH)D concentrations in female individuals, which support this possibility. As can be seen in Table 2, hypovitaminosis and vitamin D deficiency occur significantly more often in male individuals, even using a 75 nmol/L cut-off value, whereas higher serum 25(OH)D concentrations are seen in female individuals.

Despite the fact that mean serum 25(OH)D concentrations were in the reference range, 27.2% of the patients had hypovitaminosis or vitamin D deficiency. Of the healthy individuals, 26.35% had hypovitaminosis D and 2% of them had vitamin D deficiency. In particular, healthy individuals over 45 years of age and teenage girls had significantly lower mean serum 25(OH)D concentrations and attention should be paid to correcting the low serum 25(OH)D concentrations in teenage girls (Table 5) because the need for supplementation is unequivocal for reducing the risk of osteoporosis at an older age. The effect of the provision of obligatory vitamin D_3_ supplementation in infancy resulted in the highest mean serum 25(OH)D concentrations (Table 5; Figure 8).

Vitamin D deficiency and hypovitaminosis are undesirable in any disease, but they were detected in all groups of diseases. Some risk factors for a low vitamin D status have been named in a publication by Amrein et al. (2020). In our study, there are some groups in which vitamin D deficiency and hypovitaminosis D are more common and affect at least a quarter of the patients. They include certain conditions originating in the perinatal period, diseases of the genitourinary system, mental and behavioral disorders, diseases of the circulatory system, diseases of the skin and subcutaneous tissue, diseases of respiratory system symptoms, signs and abnormal clinical and laboratory findings not classified elsewhere, and diseases of the musculoskeletal system and connective tissues. Among patients with certain conditions originating in the perinatal period (P00–P96), almost 50% had hypovitaminosis or vitamin D deficiency, indicating the need for greater attention on vitamin D supplementation. In the diagnosis group of sine morbo patients (U9990), significantly more female than male patients were detected and 28.3% of them had low serum 25(OH)D concentrations. We think this is a very important finding because these patients will probably start vitamin D supplementation as prevention, indicating health-awareness behaviors. In our opinion, a clinical pharmacist should encourage such “sine morbo” people to take vitamin D supplementation. Analyzing ICD-10 in detail, we found that significantly higher odds for vitamin D deficiency and hypovitaminosis could be related to 20 of the diseases in the list; they are *in situ* neoplasms; diabetes mellitus; obesity; and organic, including symptomatic mental disorders, epilepsy, cardiovascular diseases, non-infective enteritis and colitis, papulosquamous disorders, renal failure, and certain conditions originating in the perinatal period (Table 4). Many metabolic disorders like diabetes mellitus and obesity are among them, as are gastrointestinal diseases. In diabetes mellitus, supplementation with vitamin D might reduce the risk of consequences of diabetes like diabetic neuropathy by improving microcirculation and reducing inflammation (Karonova et al., 2020). Furthermore, a research group confirmed that at least a 100 nmol/L serum 25(OH)D concentration decreases prediabetic patients’ risk of diabetes (Dawson-Hughes et al., 2020). Interestingly, there was no benefit from vitamin D supplementation in cardiovascular diseases in the literature (Nudy et al., 2020). Based on an earlier meta-analysis, vitamin D supplementation did not reduce major cardiovascular events or all-cause mortality (Barbarawi et al., 2019). In comparison, our data suggest that there is significant correlation between cardiovascular diseases and hypovitaminosis and vitamin D deficiency, as would be expected from a recent non-linear Mendelian randomization study showing marked CVD risk reduction with genetically increased serum 25(OH)D values in deficient subjects (Zhou et al., 2022). We think that our data and the publications mentioned are not contradictory. This might mean there is an extra factor of risk in a multifactorial disease, especially if we realize that diabetes mellitus and obesity are also risk factors for cardiovascular diseases and that obesity specifically reduces serum 25(OH)D values. Accumulated fat in the adipose tissue triggers inflammation through immune cell infiltration and the release of inflammatory mediators, which act as an obesity risk factor for many diseases like type 2 diabetes mellitus, cardiovascular diseases, cancer, and COVID-19 (Uribe-Querol and Rosales, 2022). Thus, obesity-related lower serum 25(OH)D concentrations could confound the studies on associations between vitamin D supplementation and cardiovascular diseases (Baker et al., 2015), which was also found with cancer (Manson et al., 2019), both such groups having a lower vitamin D status. Although cardiovascular diseases, e.g., stroke or heart attack/myocardial infarctions are sudden events, the underlying disease develops slowly, over many years. In some central nervous system diseases like epilepsy, we also found lower serum 25(OH)D concentrations, which is important since some enzyme inducer antiseizure drugs are known to cause vitamin D deficiency (Holick, 2017), so vitamin D supplementation has to be kept in mind.

The guidelines recommend different doses of vitamin D_3_ supplementation for the general population from 200 to 800 IU daily (Pludowski et al., 2018) and even daily cholecalciferol doses of 1,000–2,000 IU/day (25–50 µg/day) in case of vitamin D deficiency and insufficiency guideline failure, especially in populations with known prevalent vitamin D deficiencies (Płudowski et al., 2023). Patients suffering from a disease require higher doses of 3,000–100,000 IU/day vitamin D_3_ initially. In addition to the daily administration of vitamin D_3_ weekly, loading doses may be used in order to improve patients’ adherence.

In summary, this study confirms that increasing the consumption of vitamin D_3_ products will increase serum 25(OH)D concentrations—at the patient population level.

The general recommendations for considering vitamin D_3_ supplementation to reduce vitamin D deficiency emerging from this study are as follows: a) mean serum 25(OH)D concentrations show seasonality, and supplementation should be started in September; b) 27.23% of patients have an insufficient vitamin D status; c) male subjects have higher odds for hypovitaminosis D or vitamin D deficiency; d) healthy individuals aged over 45 years and female teenagers have significantly lower serum 25(OH)D concentrations than patients with a diagnosis; e) in certain metabolic disorders, cardiovascular diseases, and central nervous system disorders, attention should be paid to vitamin D_3_ supplementation; and f) among people with sine morbo diagnosis, clinical pharmacists may play a vital role in providing information on the importance of vitamin D.

## Conclusion

The serum 25(OH)D concentrations in more than a quarter of the investigated blood samples were below the reference range. This further reinforces the notion of vitamin D_3_ supplementation being desirable for many health-risk groups and even for healthy individuals. Efforts to improve health literacy did slightly increase vitamin D_3_ consumption and mean serum 25(OH)D concentrations. However, a major threat to health, COVID-19, dramatically raised vitamin D_3_ consumption and, in its subsequent aftermath, an increase in serum 25(OH)D concentrations. Based on our findings, it should be recommended that routine vitamin D_3_ supplementation should be started in September in order to prevent decline in October so as to reduce the risk of vitamin D deficiency in the winter months and to reduce respiratory infection risks. Our findings also highlight the importance of health education for the promotion of vitamin D supplementation.

## Limitation

The authors are aware that the study has several limitations. First, we do not have data on the mode of supplementation or on the dosage. Second, we could only use the referral diagnoses. Furthermore, our study was not representative of the whole local population, being composed of only those whose 25(OH)D values were measured as the result of a laboratory request by a doctor. The drug utilization research was based on the analysis of prescription-only medicines alone, but the other market segment, i.e., OTC medicines, showed exactly the same growth. Although not having data on all factors providing vitamin D in this cohort is a weakness, the availability of serum 25(OH)D measurements of all patients helps us in compensating for that problem. Nevertheless, the strengths of the study are the size of the examined population, the size of the sample, and the 6-year duration of the study period.

## Data Availability

The raw data supporting the conclusion of this article will be made available by the authors, without undue reservation.
